# Yellow Fever Again in Cuba*This article is simultaneously published in the Spanish language, both in Europe and America.

**Published:** 1907-10

**Authors:** A. M. Fernandez De Ybarra

**Affiliations:** New York City


### TEXAS MEDICAL JOURNAL.

Established July, 1885.

F. E. DANIEL, M. D., - - - - Editor, Publisher and Proprietor

PUBLISHED MONTHLY.—SUBSCRIPTION $1.00 A YEAR.

VOL. XXIII. AUSTIN, OCTOBER, 1907. No. 4.

The publisher is not responsible for the views of contributors.

For Texas Medical Journal.

### Yellow Fever Again in Cuba.*

BY A. M. FERNANDEZ DE YBARRA, A. B., M. D., NEW YORK CITY.

In the official report of the Superior Board of Health of the Island of Cuba, published in *Revista de Medicina Tropical,* of Havana, for the month of December, 1905, there is a "Statement of the Yellow Fever Cases That Occurred In the City of Havana from October 17 to December 12, 1905," confirmed as such by the official Commission of Infectious Diseases of that island. As stated in the report, in that summary of yellow fever cases are included four (Nos. 29, 47, 54 and 57) that appeared in the interior of the island, viz., one case in the city of Matanzas; one case in a sugar plantation called "Valiente," two miles distant from the town of Alacranes (3000 inhabitants), in the same province of Matanzas, and at about thirty-four miles to the south from the city of Matanzas, in the interior of the island; one case in another sugar plantation near the town of Real Campina, in the province of Santa Clara, close to the boundary line of Matanzas province; and one case at the sugar plantation called "Julia," also in Matanzas province, and both these two cases in the interior of the island.

But other three cases of yellow fever,—one at the city of Marianao, thirteen kilometers to the southwest from Havana; one at the town of San Jose de las Lajas, thirty miles to the southeast from Havana, and one case at the sugar plantation called "Alava,"

*This article is simultaneously published in the Spanish language, both in Europe and America.

near the town of Benaguises, in the province of Matanzas, in the interior of the island,—are not included in that official report of yellow fever cases because "it has not been possible to trace the source of infection of those three cases," as the president of the Superior Board of Health of Cuba says in his official report. Which is a very flimsy reason, indeed.

Nor has another case been included in that official report (inasmuch as they also included four other cases which occurred outside the city of Havana), and a second one it was which appeared at the sugar plantation called "Valiente," in Matanzas province.

Adding those four unreasonably excluded cases, with three deaths, to the fifty-eight cases and twenty-two deaths (twenty-one of which being marked "under treatment") of the "Statement," they form a total of sixty-two officially recognized cases of yellow fever, with twenty-five deaths, that appeared in Cuba in different localities of the western part of the island in the short period of less than two months (from October 17 to December 12, 1905), and during the beginning of the winter season of the year 1905.

To those sixty-two cases must be added the other two that appeared at Punta de Sal, near the city of Santiago de Cuba, in the eastern part of the island, at the end of the previous year, 1904, the source of their infection not having been traced, either, to yellow fever infected mosquitoes. And ten cases, with four deaths, more that occurred in the city of Havana, from December 12 to December 31, 1905, and that likewise, unreasonably, were not tabulated in the official "Statement" (which in my opinion ought to have been dated December 31, the last day of the month and of the year, instead of December 12), a total of seventy-four cases of yellow fever, with twenty-nine deaths, is reached during the years of 1904 and 1905.

Coming now to the statistics of cases in the epidemic of 1906, or, rather, the uninterrupted continuation of the same epidemic of 1905, from January 1 to July 1, 1906, we find the following official figures, which I have gathered from the published reports of the Superior Board of Health of the Island of Cuba, and the *Public Health Reports,* issued weekly at Washington, D. C., by the Surgeon General of the Public Health and Marine Hospital Service of the United States:

January 1, 1906—1 case. Died January 3.

January 16, 1906.—1 case. This case was not reported until the autopsy, made January 16, had revealed the typical pathological lesions of yellow fever.

January 20, 1906.—1 case. In this case the same circumstances occurred, that is, the diagnosis was not officially confirmed until after the autopsy was made.

January 30, 1906.—1 case.

January 31, 1906.—1 case.

February 2, 1906.—1 case.

February 6, 1906.—1 case. This case appeared in Matanzas province in the sugar plantation colony called "Atrevido," near the town of Bolondron, at about thirty-four miles south from the city of Matanzas. Died.

February 7, 1906.—1 case. This case was a Cuban lad, 16 years of age, who died.

February 11, 1906.—1 case.

May 18, 1906.—1 case. Died.

May 23, 1906.—1 case. This case occurred in Matanzas province, in the vicinity of the town of Union de Reyes, at about thirty miles south from the city of Matanzas.

June 7, 1906.—1 case. Died. This case occurred at No. 93 Muralla street, and was treated at "Covadonga" hospital.

June 12, 1906.—1 case. Died. This case occurred at the Miramar hotel, which is the most exclusive one in Havana.

June 18, 1906—1 case.

June 21, 1906—1 case.

Total, 15 cases and 8 deaths from January 1 to July 1, 1906.

So that a grand total of eighty-nine cases of yellow fever with thirty-seven deaths (a mortality of 41.57 per cent) have made their appearance in Cuba since the much-vaunted etiological theory of the female *Stegomyia fasciata* mosquito as the only cause of the development of that disease, and the exclusive means of its propagation was discovered in that very island of Cuba in 1900, proudly proclaimed there and in the United States as the future salvation from the ravages of that fearful disease, and since the arrogant defiance to the reappearance of any more epidemics of yellow fever in Havana and in New Orleans (in both of which cities yellow fever did reappear)—founded on the discovery of that theory, which was made to order, and by false analogy similar to the etiological theory of malaria—was cast to the four winds of heaven, and loudly praised and cheered in highly scientific medical journals.*

*See the article, "La propagation de la fievre jaune," by Prof. G. Sandrelli, in reference to the last epidemic in New Orleans, in "Revue d'Hygiene et de Police Sanitaire," May, 1906.

And it is a singular fact, worthy of careful consideration, that the epidemic of yellow fever in Cuba in 1905 and 1906 and 1907 made its reappearance in the western part of that island where the pretended extermination of *Stegomyia fasciata* mosquitoes has continuously been carried on since February 15, 1901 (the date of the very commencement of that prophylactic campaign *in the city of Havana only*); while the eastern part of the island, where the great and appalling epidemic of yellow fever which appeared there during the Spanish-American war, and was effectually eradicated without killing mosquitoes, nor protecting the yellow fever patients against their bites, and, also, where no active war on mosquitoes has ever since that time been carried on, nor before, has remained all the year 1905, 1906, and up to the present time, entirely free from yellow fever.

I will give now the clinical history of several of those Cuban yellow fever cases, as officially published by the Superior Board of Health of Cuba and the Public Health and Marine Hospital Service of the United States, in which cases the origin of the disease could not be traced to yellow fever infected mosquitoes, in spite of the most searching investigation, thus showing hereby with practical and irrefutable proofs the fallacy of that etiological theory of the disease. And without any further comment I will end this paper by reproducing (translated by me into English from its Spanish original) the above-referred official "Statement of the Yellow Fever Cases that Occurred in the City of Havana from October 17 to December 12, 1905," with a short resume of those cases made by me.

CLINICAL HISTORY OF YELLOW FEVER CASES FAILING TO PROVE THE MOSQUITO ETIOLOGY AND ONLY MEANS OF TRANSMISSION OF THE DISEASE.

1. *Clinical History of the Two Cases of Yellow Fever at Punta de Sal, in the Province of Santiago de Cuba.* FIRST CASE.—From an official cablegram sent by the representative of the Public Health and Marine Hospital Service of the United States at the city of Santiago de Cuba to his chief in Washington, D. C., dated October 24, 1904, the following is quoted: "The local board of health reports one case of yellow fever at Punta de Sal, has been sick seven days. Can not yet trace source of infection. Transferred him to isolated Cayo Duane (quarantine station). Issued bill of health shows this. Will write first mail.—WILSON."

On receipt of this report, via Washington, D. C., and another one from Dr. Caminero, Cuban medical officer of the port of Santiago de Cuba, the Superior Board of Health of the Island of Cuba immediately sent Dr. Juan Guiteras, as a yellow fever expert, to examine the case, and he wired that all the symptoms were those of yellow fever, yet he was not able to confirm the diagnosis already made.

From all the information received at Havana, no vessel had arrived at the port of Santiago de Cuba from any infected yellow fever place since the beginning of September, 1904, and it seemed very strange to all the sanitary authorities of Cuba that yellow fever should develop there without any cause to propagate it.

The patient, Mr. S. A. Fuller, was a white native of the United States, 24 years of age, who arrived at Santiago de Cuba on September 23, 1904, in the American steamship Orizaba from New York. He went immediately to Punta de Sal (the port of shipment of the Cobre copper mines), and afterward made several visits to the city of Santiago de Cuba, near by, across the bay. On Sunday, October 16, he was again in that city and drank beer and other drinks to excess. On Monday, October 17, he felt sick and heavy, but kept at his work as a foreman of the mines. On Tuesday, the 18th, he was compelled to quit his work on account of his sickness, but no physician was called in to see him until Thursday, the 20th, on which day Dr. Jose Bisbee saw him and found that he had a high fever, 40.2 C., pulse 92, frontal headache, rachialgia, marked epigastralgia, flushed face, bilious vomiting, epistaxis, injected eyes, great restlessness and scanty urine, but without albumin. He continued in this condition until Saturday, the 22d, when Dr. Bisbee called in consultation Dr. I. P. Agostini, chief of the municipal sanitary department of Santiago de Cuba, and on Sunday, October 23, the case was reported as one of suspected yellow fever.

The official Commission of Infectious Diseases of the city of Santiago de Cuba went that day to Punta de Sal, examined the patient and found all the symptoms as described above, and in addition a great quantity of albumin in the urine. On October 27, that is, on the tenth day of the disease, Dr. Juan Guiteras officially confirmed the diagnosis of yellow fever, but not until then.

Second Case.—The patient, Mr. Slater, was a young American, af the white race (age not given), who was assistant to the foreman, Mr. Fuller, in his work at the copper mines. He was taken sick on November 2, 1904, that is, ten days after Mr. Fuller had been removed to Cayo Duane, October 23, and the longest period of incubation of the disease (six days) had already passed. At once he was put under observation as a suspicious case of yellow fever and sent to the lazaretto at Cayo Duane. This case was officially confirmed on November 8, and not until then.

2. *The First Case of the Epidemic of 1905 at the City of Havana.*—The patient was a native of Spain, who had steadily lived in Cuba for several years, was employed as a truckman by a commercial house, and often went to the main wharf of the city (Muelle de Caballeria) to carry merchandise for his employers. He was taken sick on October 17 and died on October 25. The death certificate stated that the patient had died of a "dangerous jaundice" (ictero grave), but, upon investigation by the Superior Board of Health, it was discovered that the case had been a typical one of yellow fever. The mosquito origin of the infection could not be found.

3. *The Case at the City of Mariano, Thirteen Kilometers Southwest from Havana.*—Rosalia Fernandez, born in Spain, wet nurse to a child in the family that lives at No. 34 General Lee street. She had lived in Cuba during the last fourteen months, the final four of which had her steady residence at the above address, without having gone out of that house at all during those four months. She got sick on November 23, 1905; was taken to "Las Animas" hospital as a suspicious case of yellow fever, and on November 25 the official Commission of Infectious Diseases confirmed the diagnosis of yellow fever.

4. *The Case at San Jose de las Lajas, Thirty Miles to the Southeast from Havana.*—Francisco Lopez, born in Spain, lived in Cuba during the last three months, residing all that time in San Jose de las Lajas, in the tenement building owned by the trolley car company, and worked as a laborer on the branch of that company which extends from San Jose to the city of Guines. He asserted that he had not been in the city of Havana before getting sick. On November 26 he took to his bed with symptoms of yellow fever, was transported to "Las Animas" hospital on December 1, and the liagnosis was confirmed that very day by the official Commission of Infectious Diseases.

5. *The Case Near the Town of Banaguises, in the Province of Matanzas.*—Maria Mendez Freire, born at Lugo, Spain, arrived at Havana as an immigrant on November 14, 1905, remained twenty-four hours at the isolation station of Triscornia (the immigrants' depot), and spent two and one-half days in a cheap hotel situated at No. 29 Inquisidor street, going from there by railroad on November 20 to the sugar plantation called "Alava," located near the town of Banaguises, in the province of Matanzas, in the interior of the island. She got sick with yellow fever symptoms on December 7, was taken to the municipal hospital of the town of Colon (as there was no hospital at Banaguises), which is situated in the interior of the island, at about sixty miles to the southeast from the city of Matanzas. There she was isolated in a mosquito-proof room. The diagnosis of yellow fever was confirmed by the chief of the local health board of Colon, and also by the commission of experts sent from the city of Matanzas by the Superior Board of Health of the island, and a delegate of the same who went directly from Havana.

6. *The Case at the Sugar Plantation Colony Called "Valiente," Midway Between the Towns of Union de Reyes and Alacranes, in the Province of Matanzas.*—The patient was a Spaniard who had recently arrived at Havana from his native country as an immigrant. He was transferred, as usual, from the steamer to the isolation station of Triscornia, awaiting employment. He arrived at the Sugar plantation "Valiente" on November 29, went to work the following day feeling perfectly well; worked all that day and the following one, and was taken sick December 1. No physician was called to visit the case until the sixth day of the disease, and when the patient was in a preagonic condition.

The attending physician, noting suspicious symptoms of yellow fever, reported the case to the local health officer at Union de Reyes (a town of about 3000 inhabitants, located at a distance of thirty miles to the south from the city of Matanzas), during the evening of December 6, who went to visit the patient, at a distance of two miles from Union de Reyes, in order to verify the diagnosis, on the morning of December 7, shortly before the patient died. The provincial sanitary inspector, who resides at Matanzas city, was telegraphed for, and a post-mortem examination revealed the typical pathological lesions of yellow fever.

7. *The Case Near the Town of Real Campina, in the Province of Santa Clara.*—The patient was a Spanish immigrant, who had arrived at Havana from his native country on November 18, 1905, in excellent health. After being detained at the quarantine station of Triscornia, as it is customary, he was released, and on November 27 went by railroad to work at a sugar plantation near the town of Real Campina, which belongs to the municipal district of the city of Cienfuegos, and is located close to the boundary line between the provinces of Matanzas and Santa Clara. He got sick with yellow fever symptoms on December 5, 1905. He was first carried to the town of Real Campina, but as there was no hospital there he was transferred to the town of Colon, the same as the case from near the town of Banaguises, where the diagnosis of yellow fever was confirmed on December 11, and two days after he died, on December 13.

8. *The Case in a Sugar Plantation Near the Town of Bolondron, in the Province of Matanzas.*—The patient was a Spanish immigrant who had left the city of Havana two months previously, and had been working steadily as a laborer all that time in a sugar plantation colony called "Atrevido," situated near the town of Bolondron, in the province of Matanzas, at about thirty-five miles south from the city of Matanzas, in the interior of the island. He had not had any contact or communication with any focus of yellow fever infection. The Havana sanitary authorities made a very close investigation of all the circumstances in this case, but could not find the source of infection. In view of the impossibility of tracing the infection, the diagnosis of the disease was considered doubtful until the post-mortem findings proved it to be a typical case of yellow fever.

It is very clearly proven in all these nine cases of yellow fever, except, perhaps, one (No. 6), that the disease developed after six days' exposure to the supposed mosquito infection (granting there was such, which in several of these instances here narrated could not possibly have existed), which is the longest recognized period of incubation of yellow fever.

The exclusive mosquito theory of that disease, therefore, is hereby indicted.

Relying on that fictitious theory, the sanitary authorities of Cuba firmly believed that by preventing the hatching of mosquitoes in the city of Havana (the other cities and towns of the island not having been taken into consideration), and protecting all imported cases of yellow fever from the bites of mosquitoes, the island was perfectly safe from any more epidemics of that disease. In consequence of that false sense of security, they neglected the enforcement of the well established rules of cleanliness and general sanitation, which has a continuous history of many centuries in its favor, thus slowly but surely retrograding to the fearful unsanitary condition that prevailed at the time of the Spanish sovereignty in Cuba. And to such extent that neglect of general sanitation of the cities and towns had already gone that when the *New York Herald* sent a commission of experts to Cuba to investigate the true condition of sanitary affairs, and they revealed to the world the bad state of sanitation there, the Cuban authorities and the Cuban press denounced the great New York paper in the most scathing terms. But the reappearance of yellow fever in Cuba opened wide the eyes of the Cuban people, and hundreds of thousands of dollars were hurriedly spent in cleaning Havana and all the larger cities of the island, making also provision in the government budget for good and abundant supply of water. The Cuban sanitary authorities went to the extreme of renewing and putting in force the stringent sanitary rules of a house-to-house cleaning and disinfection in the city of Havana, which were so timely and efficiently ordered by the late General Ludlow during the first American occupation of that city.

In the modern method, now so fashionable, of eradicating an epidemic of yellow fever, all the old means of accomplishing that result are employed *plus* the prevention of mosquito hatching on the surface of stagnant water, and the protection of the isolated patients from the bites of mosquitoes. Then the good results thus obtained are hypocritically attributed *only* to the campaign against mosquitoes. Why do they not leave the yellow fever patient wherever he be found sick, thoroughly protected from the bites of mosquitoes, but without isolating him in a special hospital and without carrying out all the old prophylactic measures? If it is true that "without mosquitoes there can be no yellow fever," why do they still continue to classify yellow fever as an infectious disease, and proceed to stamp it out from a locality as such?

This unabated mystification reminds me of the casuistic resource of a fellow practitioner and friend of mine when any of his patients suffering from a soft chancre of the penis complained to him that the iodoform ordered to be dusted after disinfection of the sore made people near him stop their noses, run away from him, and give vent to very unpleasant remarks. In such cases my fellow practitioner advised his patient to put some powdered iodoform on one of their fingers and bandage it well around. The people with whom they came in contact could not then distinguish that there were two sources of iodoform smell, and, consequently, seeing the bandaged finger, would stop making unpleasant remarks.

In the officially published "Statement of Cases of Yellow Fever" of the Superior Board of Health of Cuba only fifteen deaths are recorded as having occurred in the tabulated total of fifty-eight cases, but I noticed that no less than twenty-one cases are marked "under treatment," some of them as early at the beginning of the epidemic as November 16 and 19. I have investigated the termination of those twenty-one cases and found that seven of them were fatal, making a total of twenty-two deaths in the fifty-eight cases from October 17 to December 12, 1905, a mortality of 37.93 per cent. This figure clearly indicates the virulence of the disease.*

If a non-immune person who has been inoculated by the bite of a yellow fever infected female *Stegomyia fasciata* mosquito develop the disease in due time, the natural consequence of this case of yellow fever would be that the house where the patient resides, its immediate neighborhood, some non-immune individual who may have visited his residence, or in some other way became exposed to the bite of female, and only female, *Stegomyia fasciata* mosquitoes contaminated with the poisoned blood of that yellow fever patient (already hypocritically isolated in a special hospital), would necessarily be the very factors for the subsequent appearance of more cases of the disease, if the new etiological theory of yellow fever is true.

In the reappearance of the epidemic of yellow fever in Havana have those necessary factors been verified?

No, not by any means. What has happened is precisely the contrary of it, inasmuch as the first case of the epidemic occurred at No. 14 San Miguel street, the second case developed at No. 115 Aguila street, the third at No. 98 Amistad street, the fourth at No. 537 Calzada de Jesus del Monte, the fifth at the Inglaterra hotel, on the Prado (the most fashionable street in the city), the ninth in a bark anchored in the bay, another one in the aristocratic suburb of Vedado, another one in the city of Regla, on the other side of Havana bay, another case developed in the city of Marianao, thirteen kilometers southwest from Havana; another case at the town of San Jose de las Lajas, thirty miles to the southwest from Havana; another one in the city of Matanzas, sixty miles east from Havana; another in a sugar plantation in Santa Clara province, etc., etc., etc., places all of them very far from each other.

---

*Please read my short article on "The Modern Treatment of Yellow Fever," published in "the Therapeutic Gazette," April 15, 1905, and in the Spanish language in "El Siglo Medico," of Madrid, "Gaceta Medica Catalana," of Barcelona, "Semana Medica," of Buenos Ayres, etc.

And all these cases occurred in persons who had not been in any way in contact or near one another. And even granting, for the sake of argument, that they had been exposed to the bite of contaminated female *Stegomyia fasciata* mosquitoes, the incubation period of the disease had already long passed away when they got sick.

In the case of the two Italian friends, who lived, respectively, at No. 115 Aguila street and at No. 98 Amistad street, there is no doubt that both were infected in the same locality (whatever that locality may have been); but it is certain that the second case was not infected by any female mosquito which might have bitten the first case, for the very convincing reason that the time elapsed between the sickness and death of the first patient and the sickness of the second patient does not leave room even for the suspicion that the one was infected by the other. [From Dr. Tomas Vicente Coronado's paper, entitled "Is It Possible to Confound Yellow Fever with Other Febrile States?" read by him before the Academy of Medical Sciences of Havana, Cuba, on November 24, 1905, and published in the *Anales de la Academie de Ciencias Medicas,* Vol. XLII, October-December, 1905-1906, page 214.]

The sophisticated assertion that "without mosquito there can be no yellow fever" appears in all its nude form when it is remembered that the commission that discovered the mosquito etiology and only manner of transmission of that disease, proved beyond all possibility of a doubt that yellow fever can be very easily transmitted from a sick room to a healthy non-immune by the simple use of a hypodermic syringe. And without the intervention of any mosquito whatever.

And the no less deceptive pretension that the female *Stegomyia fasciata,* and no male mosquito of the same species whatsoever, transmits yellow fever because the female needs human blood to be able to complete the physiological process of ovulation, when it is perfectly well-known that thousands of millions of female *Stegomyia fasciata* mosquitoes in the marshes and swamps go through all their existence, accomplishing to the fullest extent the physiological process of ovulation, and thus effectually reproducing their own species *without ever having had a single meal of human blood.*

Regarding the apparently formidible argument, so often used as a trump card, of the corroborative observations of the mosquito theory of yellow fever independently made in Brazil by the French commission from the Pasteur Institute of Paris, the English commission from the Liverpool School of Tropical Medicine, and the German commission from Hamburg, my explanation of the misleading results obtained by all of them is that they all are founded on exactly the same unsteady ground principle. All those eminent men of science (many of them, like in the American commission, never having seen a single case of yellow fever before), made the mistake of unwittingly repeating the fundamental experiments of the American commission at Havana in 1900, which, in my opinion, were improperly conducted under abnormal and artificial conditions, instead of following the natural process of the development of yellow fever.* In other words, they ought to have, first, studied the atmospheric and telluric conditions under which yellow fever develops, and to have made their experimental investigations in a native-built house, located in a focus of yellow fever infection, with all the unsanitary conditions there existing in a natural state (as much in regard to the house itself as regarding the town in which it was located), but thoroughly well protected against the entrance of mosquitoes, instead of building, in a healthy locality, a sort of an ideal autoclave to make the experiments in; they ought to have been extremely careful in the selection of non-immune persons to be used as test cases, which precaution the American commission failed to take properly; they ought to have confined those persons to live steadily in that native-built house, effectually contaminated with yellow fever poison, both day and night, instead of putting them in the ideal autoclave, as they did, *only to sleep,* and taking them out of it each morning to remain all day in a thoroughly healthy place, thus imitating in an inverse order Penelope's weaving,—undoing in the daytime what had been done during the night, which is exactly the same as if a person were taking a slow poison at night and an antidote for it in the daytime. They ought to have fed those non-immune persons in the same way, with exactly the same kind of food, and in the same amount that they were accustomed to be fed, instead of feasting them upon a highly nutritious, abundant and very strengthening dietary, in that way enabling their systems to better combat the depressive action of the infecting poison, whatever it

*See my critical dissertation on "The Fallacy of the Mosquito in Yellow Fever," published in the "Cronica Medica Mexicana," of the City of Mexico, for September, October, November and December, 1905; also in "El Siglo Medico," of Madrid, Spain, for November 25, December 2 and 16, 1905, January 20, February 24, March 10 and 17, 1906; in "Revista Medica," of Sao Paulo, Brazil; in "Boletin de la Asociacion Medica de Puerto Rico," San Juan, Porto Rico, and other medical journals.

may be, and to come out of the experiments (as it did actually happen, both in Cuba and in Brazil) in a much healthier and stronger condition than when they began the ordeal.

And why have they not also made, as they should, controlling experiments with biting flies, bed bugs, fleas, chigoes, sand ticks and lice?

**Official Statement of the Cases of Yellow Fever That Occurred at Havana, Cuba, From October 17, to December 12, 1905.**

| Number. | Date of sickness. | | Born in— | Place of residence. | Result. |
|---|---|---|---|---|---|
| 1 | October | 17 | Spain | 14 San Miguel street | Died October 25. |
| 2 | October | 23 | Italy | 115 Aguila street | Died October 29. |
| 3 | October | 29 | Italy | 98 Amistad street | Cured November 16. |
| 4 | November | 4 | Spain | 537 C. de Jesus del M | Cured November 23. |
| 5 | November | 5 | United States | Inglaterra Hotel | Died November 11. |
| 6 | November | 5 | Spain | 113 Compostela street | Died November 12. |
| 7 | November | 8 | Spain | 64 Amistad street | Cured November 23. |
| 8 | November | 9 | United States | 115 Industria street | Cured November 21. |
| 9 | November | 9 | United States | Bark "Alex. Black" | Cured November 21. |
| 10 | November | 10 | Spain | 62 Zulueta street | Cured November 29. |
| 11 | November | 11 | United States | Louvre Hotel | Cured November 22. |
| 12 | November | 12 | United States | 16 San Rafael street | Died November 21. |
| 13 | November | 13 | Germany | 125 Industria street | Cured December 10. |
| 14 | November | 15 | Spain | 154 Zanja street | Died November 27. |
| 15 | November | 16 | Spain | 68 Obispo street | Cured December 3. |
| 16 | November | 17 | Spain | 106 Marti street, Regla | Under treatment. |
| 17 | November | 17 | Spain | Isla de Cuba Hotel | Died November 28. |
| 18 | November | 17 | Spain | 134 Habana street | Died December 1. |
| 19 | November | 18 | England | 16 San Rafael street | Under treatment. |
| 20 | November | 18 | Spain | 8 Obrapia street | Cured November 27. |
| 21 | November | 18 | Spain | 134 Habana street | Died November 30. |
| 22 | November | 19 | Spain | 1½ S. Rafael street | Cured December 7. |
| 23 | November | 19 | United States | 93a Paseo de Marti | Cured November 29. |
| 24 | November | 19 | Spain | 134 Habana street | Cured December 8. |
| 25 | November | 19 | Spain | 75 Monserrate street | Died December 2. |
| 26 | November | 19 | Spain | 60 Neptuno street | Died November 29. |
| 27 | November | 20 | Spain | 103 Paseo de Marti | Under treatment. |
| 28 | November | 20 | Spain | 39 Banos street, Vedado | Cured December 7. |
| 29 | November | 21 | Spain | 124 Consulado street. (This case belongs to Matanzas city.) | Cured December 4. |
| 30 | November | 23 | England | 122 San Nicolas street | Cured December 3. |
| 31 | November | 24 | United States | 23 Concordia street, | Cured December 7. |
| 32 | November | 25 | United States | 23 Concordia street | Cured December 9. |
| 33 | November | 25 | Spain | 21 and 23 San Jose street | Cured December 6. |
| 34 | November | 26 | Spain | 123 Concordia street | Cured December 8. |
| 35 | November | 26 | United States | 1 Concordia street | Died December 4. |
| 36 | November | 27 | Spain | 145 San Rafael street | Cured December 9. |
| 37 | November | 27 | Spain | 110 Industria street | Under treatment. |
| 38 | November | 28 | Spain | 54 Obispo street | Under treatment. |
| 39 | November | 29 | Spain | 140 Habana street | Under treatment. |
| 40 | November | 29 | Spain | Dependientes' Hospital | Under treatment. |
| 41 | November | 29 | Spain | San Jose and Quiroga Sts | Cured December 10. |
| 42 | November | 30 | Spain | 130 San Jose street | Under treatment. |
| 43 | November | 30 | Spain | 130 Industria street | Died December 7. |
| 44 | November | 30 | Spain | 72 Factoria street | Under treatment. |
| 45 | December | 1 | Spain | 134 Habana street | Under treatment. |
| 46 | December | 1 | Spain | 31 San Rafael street, | Under treatment. |
| 47 | December | 1 | Spain | 134 Habana street. (This case belongs to Alacranes.) | Died December 7. |
| 48 | December | 2 | Spain | 174 Industria street | Under treatment. |
| 49 | December | 2 | Cuba | 6 Concordia street | Died December 8. |
| 50 | December | 3 | Spain | 122 Esperanza street | Under treatment. |
| 51 | December | 3 | Spain | 70 Aguacate street | Under treatment. |
| 52 | December | 4 | Spain | 33 Galiano street | Under treatment. |
| 53 | December | 5 | Spain | Covadonga Hospital | Under treatment. |
| 54 | December | 5 | Spain | 136 Habana street. (This case belongs to Real Campina.) | Under treatment. |
| 55 | December | 8 | Spain | 134 Habana street | Under treatment. |
| 56 | December | 9 | Spain | 95 and 97 Obispo street | Under treatment. |
| 57 | December | 10 | Spain | La Benefica Hospital | Under treatment. |
| 58 | December | 10 | Spain | 122 Compostela street | Under treatment. |

**RESUME.**

| Date. | | Cases. | Deaths. | Date. | | Cases. | Deaths. |
|---|---|---|---|---|---|---|---|
| October | 17 | 1 | | November | 21 | 1 | 1 |
| October | 23 | 1 | | November | 23 | 1 | |
| October | 25 | | 1 | November | 24 | 1 | |
| October | 29 | 1 | 1 | November | 25 | 2 | |
| November | 4 | 1 | | November | 26 | 2 | |
| November | 5 | 2 | | November | 27 | 2 | 1 |
| November | 8 | 1 | | November | 28 | 1 | 1 |
| November | 9 | 2 | | November | 29 | 3 | 1 |
| November | 10 | 1 | | November | 30 | 3 | 1 |
| November | 11 | 1 | 1 | December | 1 | 3 | 1 |
| November | 12 | 1 | 1 | December | 2 | 2 | 1 |
| November | 13 | 1 | | December | 3 | 2 | |
| November | 15 | 1 | | December | 4 | 1 | 1 |
| November | 16 | 1 | | December | 5 | 2 | 2 |
| November | 17 | 3 | | December | 8 | 1 | 1 |
| November | 18 | 3 | | December | 9 | 1 | |
| November | 19 | 5 | | December | 10 | 2 | |
| November | 20 | 2 | | | | | |
| Total. | | 28 | 4 | Total. | | 30 | 11 |

| | Cases. | Deaths. |
|---|---|---|
| General total during the first 35 days | 28 | 4 |
| General total during the following 22 days | 30 | 11 |
| Number of deaths not recorded | | 7 |
| Cases unreasonably excluded | 4 | 3 |
| At Punta de Sal at the end of 1904 | 2 | |
| Cases that occurred from December 12, 1905, to December 31, 1905 | 10 | 4 |
| Cases that occurred from January 1, 1906, to July 1, 1906 | 15 | 8 |
| Grand total of cases and deaths up to July 1, 1906 | 89 | 37 |

These eighty-nine cases of yellow fever, with thirty-seven deaths (mortality 41.57 per cent), which made their reappearance in Cuba up to July 1, 1906, and a great many more after the first six months of that year and up to the end of the first eight months of 1907, since the supposed rigorously scientific and *infallible* manner of preventing the introduction of that dreadful disease into a locality, or its propagation after its introduction, was discovered in the year 1900, are the cases officially reported as such by the sanitary authorities of that island. But an epidemic of dengue fever was raging in Cuba at the same time, the symptoms of which closely resemble at the beginning those of yellow fever, and consequently many cases of the latter may have been mistaken for the former disease, as it was conclusively proved in two or three instances when the autopsy revealed the error in diagnosis.*

| | |
|---|---|
| Total number of reinfected streets in the city of Havana up to July 1, 1906 | 34 |
| Total number of streets with 6 houses infected | 1 |
| Total number of streets with 5 houses infected | 1 |
| Total number of streets with 4 houses infected | 2 |
| Total number of streets with 3 houses infected | 3 |
| Total number of streets with 2 houses infected | 5 |
| Total number of streets with 1 house infected | 22 |
| Total number blocks of houses in which 3 houses were infected | 2 |
| Total number blocks of houses in which 2 houses were infected | 4 |
| Total number blocks of houses in which 1 house was infected | 46 |
| Total number of blocks of houses reinfected | 52 |
| Total number of foci of reinfection in the city of Havana up to July 1, 1906 | 60 |

In one house, after five successive fumigations with burning sulphur for the extermination of mosquitoes, on account of five previous cases of yellow fever, *one more case still occurred, that is, six cases.* That house was No. 134 Habana street.

In four houses, after one fumigation with burning sulphur for the extermination of mosquitoes, on account of a case of yellow fever, *one more case still occurred, that is, two cases.*

In Concordia street, after a thorough fumigation of the whole block, on account of two cases that developed at No. 23, one on the 24th and the other on the 25th of November, 1905, no less than five other cases made their appearance at Nos. 1, 6, 20, 21 and 123.

In fifty-five houses there was only one case of yellow fever in each.

---

The Apostle of Purity.—Dr. C. A. Bryce resents the imputation that the independent medical journals are controlled by their advertising patronage, and in a forceful editorial in the August number of *The Southern Clinic* throws back the charge

*Please consult my paper, *Contribution to the Diagnosis of Yellow Fever*, read by me before the Orleans Parish Medical Society, New Orleans, and published in "The Medical News" of Philadelphia, March 26, 1877. I published it also in Spanish in several medical journals.

in the following vigorous language. He says: "Dr. Simmons, of the *J. A. M. A.*, seems to harbor a peculiar dislike to antikamnia and its compounds, and in its writeup in his pamphlet, sent gratuitously far and wide, he appeals pathetically to all physicians and journalists to leave the evil thing alone. He doesn't see how any self-respecting journalist can carry the advertisement of antikamnia, nor how any physician 'with a particle of self-respect or manhood' can continue to support any such journal.

"*The Southern Clinic* carries the advertisement of antikamnia and caries a large and influential list of subscribers—men who are neither renegades, proselytes nor ringsters—men who are, to say the least, as honorable, self-respecting and truthful as this Apostle of Purity who see-saws like a supplejack between truth and policy. We carry the antikamnia advertisement because we know from many years' experience that it will do just what we expect antikamnia to do. We know its effects, its dose, and that it is safe to administer in doses we have proven. We have found it a valuable remedy, and, as such, use it largely in a variety of diseases. We have never had any bad results from its use in large or small doses in old or young. We, therefore, carry it as an advertisement and prescribe it in practice because we *know what we are doing*, and can confidently recommend it to our professional brethren. We presume they likewise, with all due respect for themselves and honesty of purpose, prescribe antikamnia and support the *Clinic* because they know that they are both good and honest productions. Moreover, as we are relying upon our own knowledge and responsibility, we prefer *our* opinion to that of the worthy Simmons."

---

One should inquire carefully for the history of the application of carbolic acid to a wound, especially of the finger or toe, when a gangrene with a distinct line of demarkation has developed.—*American Journal of Surgery.*

---

An exquisitely tender swelling situated just above the sterno-clavicular articulation may be due to the perforation of the esophagus by a foreign body. If there are evidence of acute laryngitis, with edema of the arytenoid cartilages, the cause may be a perichondritis of one of the tracheal rings or the cricoid cartilage.—*American Journal of Surgery.*

